# RE: Contribution of Fecal Calprotectin and Fecal Immunochemical Tests to the Evaluation of Patients with Ulcerative Colitis

**DOI:** 10.5152/tjg.2024.235102

**Published:** 2024-06-01

**Authors:** Tingpeng Hu, Zhimei Zhang, Fusheng Song, Wenguang Zhang, Jun Yang

**Affiliations:** Department of Gastroenterology, Banan Hospital of Chongqing Medical University, Chongqing, China

First of all, we would like to thank Yakut^1^ for her comments on our study titled “Evaluation of Mucosal Healing in Ulcerative Colitis by Fecal Calprotectin vs. Fecal Immunochemical Test: A Systematic Review and Meta-analysis.”^2^ In response to the questions raised, the replies are as follows.

The main purpose of this study was to find a more suitable non-invasive detection method for evaluating mucosal healing in ulcerative colitis, and to determine the reaction of mucosal healing as the ultimate goal; Diagnostic efficacy is only used to reflect the method of evaluating the result, and is not an aid to diagnosis.

As for the effects of tumor, infection, hormone, time, and different individuals, the results included in this study were adjusted by covariates, and the results of fecal calprotectin and fecal immunochemical tests reflected mucosal healing after the influence of the above factors, rather than ignoring their influence on the results.

In all the studies included in this paper, the endoscopic scoring system is used as the reference standard for mucosal healing evaluation to further evaluate the degree of mucosal healing reflected by fecal calprotectin (FC) and fecal immunochemical tests (FIT). As non-invasive tests, they are of a certain reference value, and the significance of endoscopic and histological healing in determining the degree of mucosal healing is not ignored.

In addition, a total of 37 studies with 3541 patients were included in this study by method of meta-analysis. Based on a large number of previous studies, it was concluded that FC and FIT can reflect the degree of ulcerative colitis (UC) mucosal healing to a certain extent and are non-invasive, providing clinicians with improved reference but not absolute guidance.

As stated by the authors, diagnostic thresholds are not readily available because FC and FIT values can be influenced by many factors, so it is suggested that the use of this biomarker to determine mucosal healing in specific patients may be more appropriate for treatment decisions.

Thanks to Yakut^1^ for her views and comments on this article.^2^
